# Genetic Deletion of KLHL1 Leads to Hyperexcitability in Hypothalamic POMC Neurons and Lack of Electrical Responses to Leptin

**DOI:** 10.3389/fnins.2021.718464

**Published:** 2021-09-09

**Authors:** Paula P. Perissinotti, Elizabeth Martínez-Hernández, Yungui He, Michael D. Koob, Erika S. Piedras-Rentería

**Affiliations:** ^1^Cell and Molecular Physiology Department and Neuroscience Division of the Cardiovascular Research Institute, Loyola University Chicago, Maywood, IL, United States; ^2^Institute for Translational Neuroscience and Department of Lab Medicine & Pathology, University of Minnesota, Minneapolis, MN, United States

**Keywords:** leptin, Kelch-like 1, Ca_V_3.1, T-type calcium channels, POMC

## Abstract

Kelch-like 1 (KLHL1) is a neuronal actin-binding protein that modulates voltage-gated calcium channels. The KLHL1 knockout (KO) model displays altered calcium channel expression in various brain regions. We analyzed the electrical behavior of hypothalamic POMC (proopiomelanocortin) neurons and their response to leptin. Leptin’s effects on POMC neurons include enhanced gene expression, activation of the ERK1/2 pathway and increased electrical excitability. The latter is initiated by activation of the Jak2-PI3K-PLC pathway, which activates TRPC1/5 (Transient Receptor Potential Cation) channels that in turn recruit T-type channel activity resulting in increased excitability. Here we report over-expression of Ca_V_3.1 T-type channels in the hypothalamus of KLHL1 KO mice increased T-type current density and enhanced POMC neuron basal excitability, rendering them electrically unresponsive to leptin. Electrical sensitivity to leptin was restored by partial blockade of T-type channels. The overexpression of hypothalamic T-type channels in POMC neurons may partially contribute to the obese and abnormal feeding phenotypes observed in KLHL1 KO mice.

## Introduction

The KLHL1 protein belongs to a family of actin-organizing proteins related to Drosophila Kelch that is expressed primarily in brain tissues, including the cerebral cortex, hippocampus, thalamus, hypothalamus and cerebellum (Nemes et al., 2000; Chen et al., 2008). KLHL1 mRNA is also highly expressed in hypothalamic centers associated with the regulation of energy balance, such as the ventromedial (VMH) and arcuate (ARC) nuclei (Chen et al., 2008). KLHL1 protein modulates voltage-gated calcium channel function among other effects (He et al., 2006). KLHL1 enhances Ca_V_3.2 T-type function by maintaining the channel at the membrane, it directly interacts with the α_1__H_ subunit of the Ca_V_3.2 channel and promotes its reinsertion into the membrane from the recycling endosome (Aromolaran et al., 2009).

Acute downregulation of KLHL1 using shRNA results in reduced calcium current densities and altered synaptic properties in cultured hippocampal neurons (Perissinotti et al., 2014). KLHL1 KO mice display similar alterations but they also exhibit compensatory expression to sustain calcium current homeostasis in hippocampal neurons, yet they still display synaptic alterations (Perissinotti et al., 2015). Moreover, DRG neurons from KLHL1 KO mice exhibit and uncompensated reduction of Ca_V_3.2 expression, resulting in reduced neuron excitability and decreased sensitivity to pain (Martínez-Hernández et al., 2020). Thus, deletion of KLHL1 results in differential alteration of calcium currents and neuronal excitability depending on their role in specific nervous system areas.

We recently reported that T-type channels are integral to the signaling cascade that mediates increased excitability in POMC neurons induced by leptin (the Jak2-PI3 kinase-PLCγ pathway). T-type channels (Ca_V_3.1 or Ca_V_3.2) exist in a macromolecular complex with TRPC1/5 channels. TRPC1/5 channel activity is recruited by PLCγ, generating membrane depolarization, T-type channel-initiated action potentials and increased excitability (Qiu et al., 2010; Perissinotti et al., 2021).

Here we explored whether genetic ablation of KLHL1 leads to alterations in T-type channel function in the KLHL1 KO hypothalamus. We first assessed the expression levels of TRPC and calcium channels, important targets in the feeding control centers. We found that genetic elimination of KLHL1 resulted over-expression of T-type Cav3.1, increased T-type channel function and neuronal excitability in cultured POMC neurons. Although signaling to the nucleus and Akt/mTOR signal transduction by leptin appear to be normal, the over-expression of Cav3.1 channels circumvented the effect of leptin on POMC neuron excitability, as their basal excitability was already augmented. Leptin sensitivity was restored after partial block of T-type channels with NNC-550396. KLHL1 KO mice displayed adult obesity and abnormal re-feeding after overnight fasting. This work further highlights the importance of T-type channels on the leptin signaling cascade and POMC neuron excitability; abnormally basal hyperexcitability may contribute to the uncharacteristic responses to orexigenic and/or anorexigenic signals observed in KLHL1-KO mice.

## Materials and Methods

The animal protocols used in this study were reviewed and approved by an independent Institutional Animal Care and Use Committee (IACUC 2016032). KLHL1-knockout (KLHL1-KO) mice were generated in the Laboratory Medicine and Pathology from the University of Minnesota (He et al., 2006). Mixed background WT and KLHL1-KO mice (129S1/Sv-Oca2 C Tyr C Kitl C C57BL/6) and EGFP-POMC^+^ mice [129S1/Sv-Oca2 C Tyr C Kitl C C57BL/6-Tg (Pomc-EGFP)1Low/J] (The Jackson Laboratory, RRID:IMSR_JAX:009593) were fed *ad libitum* on standard pellet diet. No exclusion criteria were predetermined. Altogether, 15 newborn pups were used for hypothalamic cultures and 34 adult mice (male) for fasting experiments. The study was not pre-registered. Experiments were conducted in the afternoon.

**Fasting experiments:** Adult male mice (25–31 g, 18–21 weeks old) were housed with *ad libitum* access to both food and water in a controlled environment at 21.5–22.5°C and 12 h light/dark cycles. Experimental manipulations were implemented following IACUC-approved protocols. Mice (WT, *N* = 19; KO, *N* = 15) were fasted with access to water for 20 h (experiments were started at 2 PM) prior to *ad libitum* food access. Pre-fasting, post-fasting and hourly weight after food reintroduction was monitored; weight gain and food consumption were also examined after reintroduction of food.

**Cell Culture:** Newborn pups (P0, no sex determination) were killed by decapitation after cold-induced anesthesia to abolish perception of pain. Their brains were rapidly removed and whole hypothalami were dissected and cultured as described (Perissinotti et al., 2014) and plated at a density of 25,000–35,000/coverslip and kept in a 5% CO_2_ humidified atmosphere at 37°C. Each culture was generated from two independent mice (electrophysiology data was generated from at least three independent batches of cultures, *N* = 6 mice).

**Biochemistry:** All standard reagents were obtained from Sigma (St. Louis, MO). Crude fractions were extracted from WT or KLHL1-KO whole hypothalamus using standard protocols (Florio et al., 1992). Primary antibody dilutions used: α_1__G_ (Ca_V_3.1), 1:1000 (Millipore, CA); α_1__H_ (Ca_V_3.2), 1:2,000 (Sta. Cruz Biotechnology, CA); TRPC1 (1:200) and TRPC5 (1:200) (Alomone, Israel); Phospho-Stat3 (pSTAT3), 1:500 (Cell Signaling Technology); phosphoinositide 3 phosphate (PI3K), 1:1000 (Cell Signaling Technology); phospho-PI3K (pPI3K), 1:1000 (Cell Signaling Technology); Leptin Receptor (LepRb), 1:1000 (Neuromics). GAPDH, 1:3000 (Enzo Life Science, NY) was used as internal reference to normalize for protein loading unless otherwise noted. Horseradish peroxidase (HRP)-conjugated secondary antibodies were used (1:10,000; Pierce) and developed with Supersignal Femto or West Dura (Pierce, IL) before analysis with a UVP Bioimaging Epichemi3 system (Upland, CA, United States). When necessary, Restore Western Blot Stripping Buffer (Pierce, IL, United States) was used to re-probe membranes.

**Immunocytochemistry (IC):** WT or KLHL1-KO neurons at 9-11 DIV were prepared as described (Perissinotti et al., 2014). Primary antibody dilutions: Ca_V_3.1 1:200 (Alomone) and 1:50 (Millipore); Ca_V_3.2 1:5 (supernatant, NeuroMab) and 1:200 (Sta. Cruz); POMC 1:200 (Novus); agouti-related peptide (AgRP), 1:200 (Sta. Cruz). Secondary antibodies: Alexa-594 and –647, 1:2,000 (Molecular Probes, Eugene, OR, United States). Image acquisition was done using a Multiphoton Leica TCS SP5 microscope and analyzed with ImageJ freeware (NIH) (Schneider et al., 2012).

**Electrophysiology:** Whole-cell patch clamp recordings were performed from WT or KLHL1-KO cultured hypothalamic neurons from 8-10 DIV. Hypothalamic neurons were recorded in external solution containing (in mM) 135 NaCl, 5 KCl, 2 CaCl_2_, 1 MgCl_2_, 10 HEPES, 10 glucose and intracellular solution containing (in mM) 110 K-gluconate, 20 KCl, 2 MgCl_2_, 1 EGTA, 10 HEPES, 2 ATP-Mg, 0.25 GTP-Li and 10 phosphocreatine-Tris. Pipette resistances were 3.2–4.5 MΩ. Cells with series resistance (*R*_*s*_) < 20 MΩ were used; *R*_*s*_ was compensated online (>80%). Data was acquired with and analyzed with pClamp 10 software (Molecular Devices). Cell capacitance was measured as described (Perissinotti et al., 2015). Drugs: NNC-550396 dihydrochloride and leptin were purchased from Tocris (Bristol, United Kingdom).

*Current-Voltage Relationships* (I–V Curves) were performed as described in Perissinotti et al. (2015) and Bean (1985). Briefly, currents were elicited from a holding potential (V_H_) = −90 mV (or –50) and depolarized for 150 ms to a test potential (V_T_) = −70 to + 60 mV, in 10 mV increments.

*Current kinetics*: Current activation and inactivation were assessed by stepping the neuron from V_H_ = −90 mV to V_T_ = −50 to −20 mV for 150 ms. The rate of activation was estimated from the current rise time from 10 to 90% of its maximum value (rise time 10–90%); these measurements were favored over the calculation of the time constant of activation (τ_on_) to minimize the contribution of the inactivation process on the activation rate value. Time constant of inactivation (τ_off_) was obtained from mono-exponential fits. Current deactivation τ was measured by fitting with a mono-exponential function the decaying phase of tail currents elicited from V_H_ = −90 mV to V_T_ = −30 mV and back to V_T_ = −60 to −140 mV.

*Steady-state analysis*: Steady-state activation (SSA) was analyzed with protocols stepping from V_H_ = −90 (or –50) mV to V_T_ = −90 to 0 mV (ΔV = 10 mV) for 12 ms followed by repolarization to –100 mV to evoke inward tail currents. Data were fitted by a single Boltzmann function of the form I_max_/[1 + exp ^(V^50^–V)/^*^*k*^*] + m, where I_max_ is maximal current, V_50_ is half-voltage of activation, *k* is slope factor, and m is baseline. Steady-state inactivation (SSI) was determined by stepping the membrane potential to various pre-pulse voltage levels (V*_*pre*_* = −110 to 0 mV, 1V = 10 mV) for 1 s before depolarization to a fixed test level (–30 mV) to evoke channel opening. The resulting data were also fitted to a Boltzmann function.

The following equation was used to calculate the theoretical steady-state current (I_stst_): I_stst_ = G (V_H_) ^∗^ I/I_max_ (V_H_) ^∗^ (V_H_ –50 mV), where I_stst_ is the current in the steady-state or “window current,” G is the conductance at holding voltage V_H_ that was obtained experimentally using the state activation (SSA) protocol and adjusted to a Boltzmann equation (not normalized), I/I_max_ is the fraction of T-type channels available at holding voltage V_H_ that was obtained experimentally from the steady state inactivation (SSI) protocol and adjusted to a Boltzmann equation, and 50 mV is the estimated reversal potential for calcium (Bijlenga et al., 2000; Lambert et al., 2014; Rivero-Echeto et al., 2021). Reversal potential for Ca^2+^ ions (E_Ca_) was calculated from current-voltage (I-V) curves.

*Recovery from inactivation*: T-type channel recovery from inactivation was studied with a paired-pulse protocol. T-type current was inactivated with a 400 ms pulse to −30 mV; a second 50 ms pulse to −30 mV was applied to assess recovery after a variable recovery period at V_H_ = −90 mV. Data was plotted as recovery time *vs.*% recovery; values were fitted with a double exponential function and a weighted tau (τ_W_) was calculated according to: τ_W_ = A^∗^τ_1_ + B^∗^τ_2_, where A and B are preexponential values that refer to the fraction of channels that is recovered with a given τ; A + B = 1.

*Calcium influx*: Calcium influx was determined by the current integral evoked by an action potential (AP). The AP waveform consisted of a digitized action potential with a resting potential of −70 mV, an upstroke of 116 mV/ms to a peak voltage of +50 mV, followed by a repolarizing slope at −65 mV/ms to a hyperpolarizing potential of −90 mV. The repolarization from after-hyperpolarization slope was 0.78 mV/ms to resting conditions. In a set of experiments (WT or KO-like ramps experiments) the action potential was preceded by a rate of membrane depolarization and a rheobase similar to that recorded in current clamp configuration in WT or KLHL1-KO neurons in response to a ramp rate of 20 pA/s. Influx normalization by capacitance was not necessary as it produced similar results as without normalization. The LVA component was isolated by the subtraction method; these protocols were applied from V_H_ of −50 or −90 mV prior to clamping the voltage at −70 mV for 10 ms before the triggering of the AP, so no changes in the driving force should be expected.

*Resting membrane potential (RMP)*: RMP was recorded in continuous trace mode without current injection for 20 s and averaged; voltages were corrected for liquid junction potentials.

*Spontaneous burst analysis*: RMP was recorded for 10 min. and spontaneous APs were identified for burst analysis using the Poisson surprise algorithm with a minimum burst surprise value of 5 to obtain the Poisson surprise value, burst length, spike number (*n* > 3), intra-burst frequency and mean intra-burst interval (Grace and Bunney, 1984; Legendy and Salcman, 1985; Sachdev et al., 1991; Wichmann and Soares, 2006).

*Input resistance*: Whole cell input resistance (*R*_*in*_) was determined after setting the membrane potential to −60 mV in response to a −60 pA current step.

*Membrane excitability*: AP discharges were triggered by 3 consecutive depolarizing ramps at rates of 20, 40m and 60 pA/s (1.5 s duration) from a presetting membrane potential of −75 mV (Balasubramanyan et al., 2006; Lu et al., 2006). The rheobase was determined as the minimum amount of current required for firing an action potential.

*Shape of single AP*: APs were evoked from a presetting voltage of −50 mV (to avoid T-type current activation) by a 3 ms-long rectangular pulse in steps of 50 pA.

### Statistical Analysis

These experiments were exploratory with no pre-determined endpoint and no data was excluded from the analyses. No blinding, no randomization, and no sample size calculation were performed. All measurements were done in at least three independent cell cultures. Results are presented as mean ± SEM. The parameter “*n*” indicates number of cells unless otherwise stated and “N” the number of animals. No test for outliers has been applied. The normality of data distribution was assessed by the Kolmogorov-Smirnov test. Statistical analysis was performed with the Sigma Plot 11 Software. Statistical significance (*P* < 0.05) was determined using either one/two-way analysis of variance (ANOVA) with Fisher LSD’s *post hoc* test for comparisons between multiple groups or an independent samples *t*-test for comparisons between two groups. Statistical significance (*P* < 0.05) for two proportions was determined using the Z-test.

## Results

### KLHL1 Deficiency Leads to Up-Regulation of Ca_V_3.1 α_1__G_ T-Type Channels in the Hypothalamus

**FIGURE 1:**
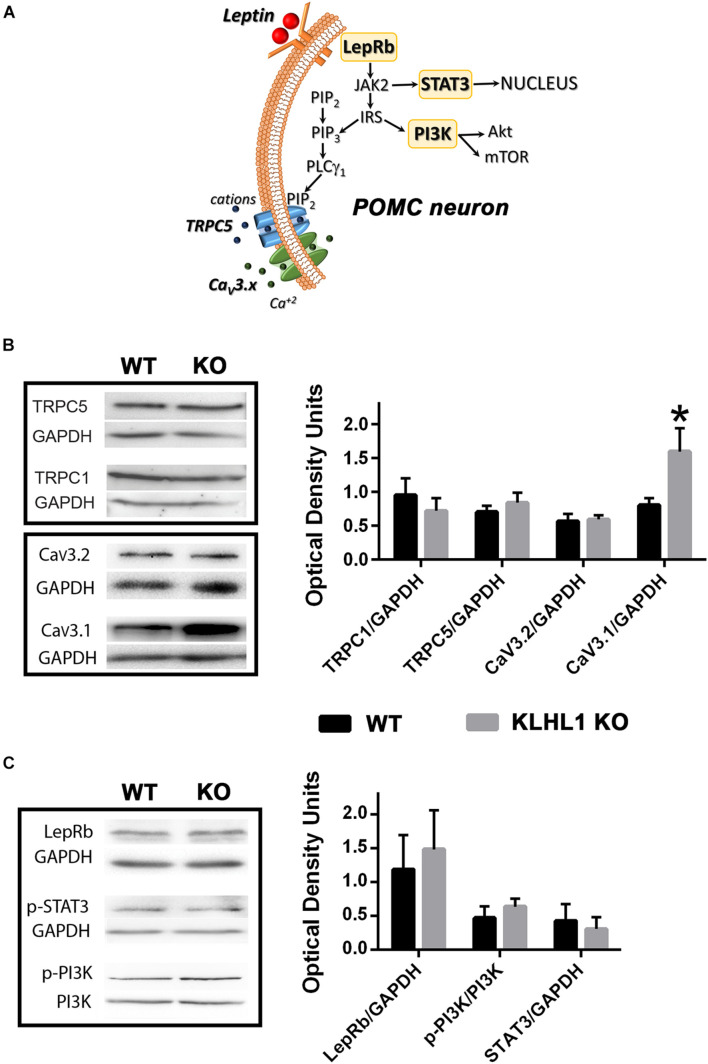
Overexpression of Ca_V_3.1 T-type calcium channels in KLHL1-KO mice hypothalamus. **(A)** Pleiotropic signaling of leptin in POMC hypothalamic neurons. Leptin binding to its receptor LRb results in the downstream activation of JAK2. STAT3 is subsequently phosphorylated and mobilized to the nucleus resulting in *de novo* gene expression. Activation of insulin receptor substrate (IRS) stimulates Akt- and mTOR pathways *via* activation of phosphorylated PI3K and the production of PIP3. The activation of PLCλ1 by PIP3 mediates the activation of TRPC1/5 channels, recruiting T-type CaV3.1/2 channels thought membrane depolarization. **(B)** Western blot analysis from hypothalamic extracts assessing TRPC1, TRPC5, Ca_V_3.2 and Ca_V_3.1 channel levels; Ca_V_3.1 protein levels are doubled in KLHL1-KO hypothalamus. Bars show densitometric quantification normalized to GAPDH. Data represent means ± S.E.M (TRPC1, *n* = 3 each; TRPC5, *n* = 4 each; Ca_V_3.2, *n* = 5 each; Ca_V_3.1, *n* = 5 each). *P < 0.05, *t*-test, *t* = 2.230, df = 8. **(C)** Western blot analysis from hypothalamic extracts assessing LRb, pPI3K and STAT3 levels. Bars show densitometric quantification normalized to GAPDH or PI3K. Data represent means ± SEM (*n* = 4 each; pSTAT3 *n* = 3 each).

The hypothalamus plays an important regulatory role in feeding, TRPC1/5 and Ca_V_3.1/2 (α_1__G_/α_1__H_) channels are crucial components in the signal transduction pathway induced by leptin that leads to neuronal excitability (Figure 1A; Qiu et al., 2010; Perissinotti et al., 2021). We explored the expression levels of these channels in the KLHL1-KO hypothalamus, as this mice model displays compensatory changes in calcium channel expression and excitability in other brain areas. As seen in Figure 1B, no changes in TRPC1/5 levels were observed in the KLHL1 KO compared to WT (*P* > 0.05, *t*-test). However, KLHL1-KO mice displayed a considerable increase in Ca_V_3.1 α_1__G_ channel expression and no statistical changes in the levels of Ca_V_3.2 (Figure 1B, *P* < 0.05, *t*-test).

We next explored other key leptin signaling molecules to assess additional leptin intracellular signaling pathways (Figure 1C). We probed the expression levels of LRb receptor, as well as STAT3 and PI3K phosphorylation levels as reporters of non-electrical signaling pathways of leptin such as signaling to the nucleus and Akt/mTOR pathway activation (Buettner et al., 2006; Williams et al., 2011). No changes in leptin receptor levels or its downstream effectors were observed in the KLHL1-KO compared to WT hypothalamus (Figure 1C, *P* > 0.05, *t*-test).

### Larger T-Type Channel Expression in KLHL1-KO Leads to Increased T-Type Current Density

**FIGURE 2:**
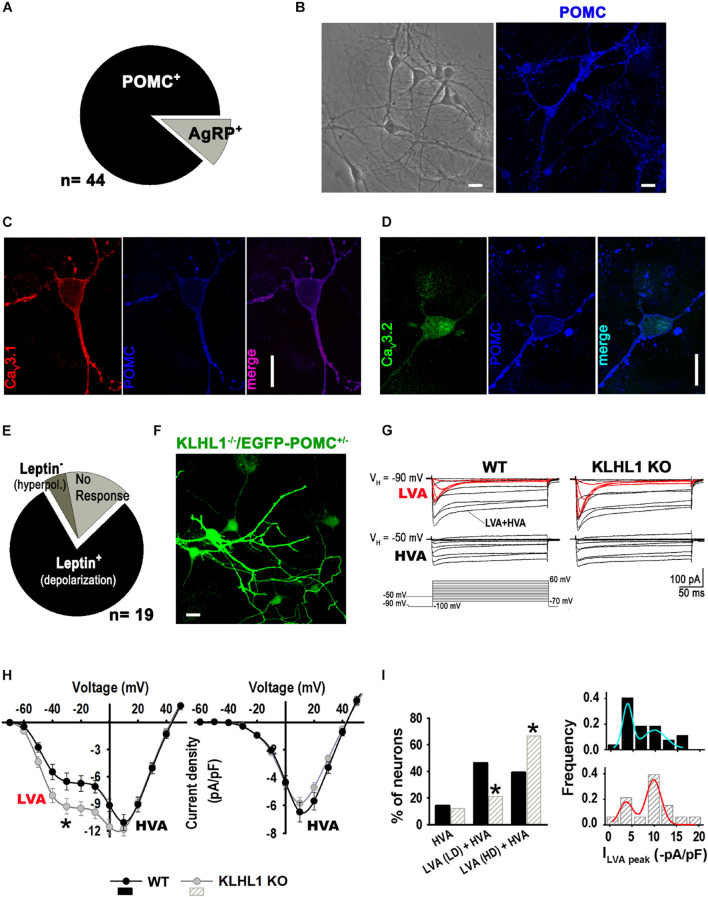
*Up-regulation of T-type current density in KLHL1-KO hypothalamic neurons.***(A)** Distribution of POMC^+^ and AGRP^+^ neurons in culture (the total neuron number was not assessed). **(B) Left:** Bright-field image of cultured hypothalamic KLHL1-KO neurons at 10 DIV. **Right:** Example of confocal image showing positive POMC^+^ neurons (blue). **(C,D)** Detection of T-type α_1__G_ (Ca_V_3.1, red) and T-type α_1__H_ (Ca_V_3.1, green) in POMC neurons. Bar size, 20 μm. **(E)** Summary of electrophysiology responses in KLHL1 KO hypothalamic cultured neurons. **(F)** Detection of POMC^+^ neurons using neurons from KLHL1 KO mice crossed-bred with mice displaying EGFP^+^ expression driven by the POMC promoter. **(G)** Average current traces obtained using the square voltage protocols shown. LVA refers to low-threshold activated calcium currents (T-type, Ca_V_3 channels). HVA refers to high-threshold activated calcium channels. **(H)** Average current-voltage relationship (I-V curve) profiles in WT (*n* = 26) and KLHL1-KO (*n* = 28) hypothalamic neurons obtained from V_H_ of –90 mV (left) or a V_H_ of –50 mV (right), *P < 0.05 (from –60 to 0 mV), *t*-test; for –30 mV: *t* = 2.183 (df = 52). **(I) Left:** Comparison of the relative contribution of each neuronal group found: HVA, neurons expressing only high-voltage activated current; LVA (LD) + HVA, neurons expressing HVA and low LVA current density levels (LD, I_LVA peak_ < 6 -pA/pF); and LVA (HD) + HVA, neurons expressing HVA and high LVA current density (HD, I_LVA peak_ from 6 to 20 -pA/pF). *P < 0.05, Z- test. **Right:** LVA current density histogram fitted with superimposed Gaussian functions.

Enhanced T-type channel expression could lead to abnormal oscillatory responses; thus, we characterized and assessed the impact of increased hypothalamic Ca_V_3.1 protein expression on POMC neuron function and their electrical responses to leptin, which positively regulates these neurons. Cultured hypothalamic neurons were studied from 8-10 days *in vitro* (DIV). Immunocytochemistry analysis from KLHL1-KO (KLHL1^–/–^) cultures yielded similar results to our previous analysis of WT neurons (Perissinotti et al., 2021). From a total of 44 neurons, the distribution of POMC^+^ *vs*. AgRP^+^ neurons was 89 *vs*. 11% (*P* < 0.05, Z-test, Figure 2A). Figure 2B shows a representative bright-field microscopic image of cultured hypothalamic neurons and a representative confocal ICC image, depicting a cluster of POMC-positive neurons in an area of comparable size. Figures 2C,D corroborate the expression of Ca_V_3.1 and Ca_V_3.2 in KLHL1-KO POMC neurons (no changes in the cellular distribution of channels were observed among WT and KO neurons).

We analyzed the response to leptin in a subset of WT pyramidal neurons using electrophysiology (other neuronal morphologies were not explored); from a total of 19 neurons, 79% responded positively to leptin (excitable), 5% were inhibited and 16% did not respond (Figure 2E), as we previously reported (Perissinotti et al., 2021). Neuron cultures from KLHL1^–/–^/EGFP-POMC^+/–^ mice were used to verify the identity of POMC neurons (Figure 2F). Ca^2+^ current properties from WT and KLHL1-KO neurons are shown in Figure 2G; average traces of low-voltage activated (LVA) and high-voltage activated (HVA) currents were elicited by depolarizing steps from V_H_ = −90 or −50 mV, respectively. Note that LVA currents (red traces) were larger in KLHL1-KO neurons compared to WT, whereas no changes were observed in the HVA component. Overall, total T-type current density was 40% larger in KLHL1-KO neurons (6.2 ± 0.9 *vs.* 8.7 ± 0.8 pA/pF at −30 mV, *P* < 0.05, *t*-test) (Figure 2E), corrobating a functional increase in these currents as suggested by the increased protein levels (Figure 1B). We defined three distinct groups of neurons according to their I-V curves: neurons that expressed only high-voltage activated (HVA) current (I_LVA peak_ = 0 pA/pF in the histogram in Figure 2H, right), neurons that expressed HVA and low LVA current density levels (LD, I_LVA peak_ < 6 -pA/pF) and neurons that expressed HVA and high LVA current density (HD, I_LVA peak_ from 6 to 20 -pA/pF). The incidence of neurons with HD LVA was much larger in KLHL1-KO neurons (66.7% *vs.* 39.3% in WT; *P* < 0.05, Z-test; Figure 2I).

**FIGURE 3:**
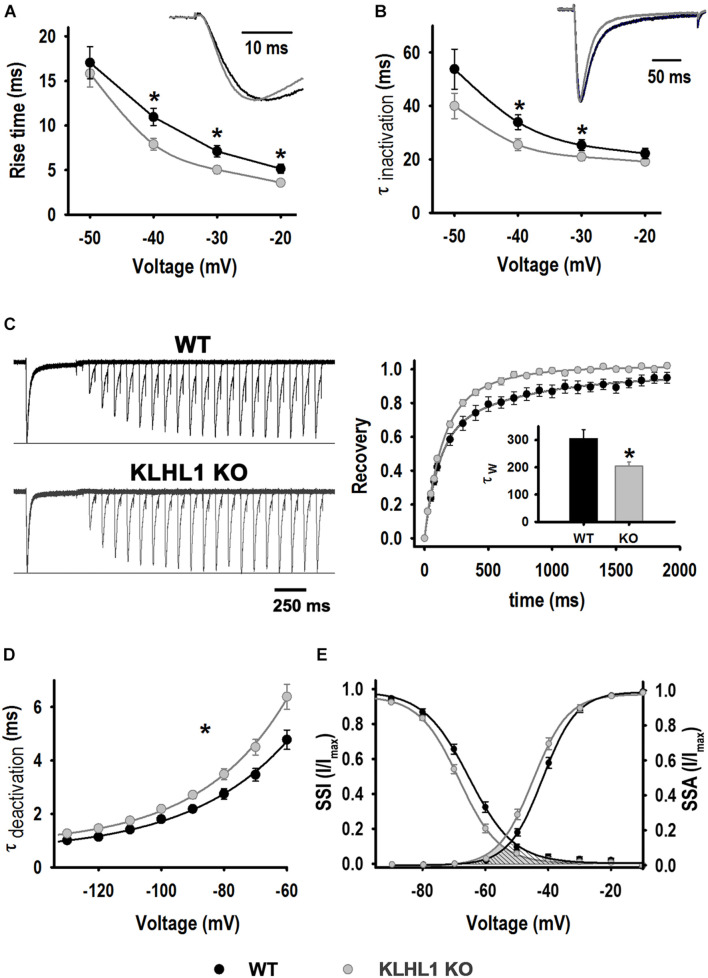
LVA current properties. **(A)** Activation kinetics. Time taken by the current to change from 10% to 90% [Rise time (10–90%)] as function of voltage for WT (*n* = 21) and K-LHL1-KO (*n* = 26) neurons. *P < 0.05, *t*-test, for –40 mV: *t* = 2.723 (df = 45), for –30 mV: *t* = 2.743 (df = 45) and for –20 mV: *t* = 2.735 (df = 45). **(B)** Time constant of inactivation (τ_inactivation_) as function of voltage for WT (*n* = 21) and KLHL1-KO (*n* = 25) neurons. *P < 0.05, *t*-test, for –40 mV: *t* = 2.432 (df = 45), for –30 mV: *t* = 1.807 (df = 45). **(C) Right:** Trace examples of recovery rate from inactivation. **Left:** % recovery *vs.* recovery time (*n* = 10 for WT and *n* = 11 for KLHL1 KO). Inset: Recovery time constant (τ_W_) values. *P < 0.05, *t*-test, *t* = 2.719 (df = 19). **(D)** Time constant of deactivation (τ_deactivation_) for WT (*n* = 16) and KO (*n* = 23) neurons. *P < 0.05 at all voltage tested, *t*-test: *t* = 2.515 (–60 mV), *t* = 2.488 (–70 mV), *t* = 2.419 (–80 mV), *t* = 2.666 (–90 mV), *t* = 2.405 (–100 mV), *t* = 2.757 (–110 mV), *t* = 2.837 (–120 mV), *t* = 2.433 (–130 mV), df = 37. **(E)** SSA and SSI. SSA values obtained for WT: V_50_ = –41.8 ± 0.7 mV and *k* = 5.2 ± 0.3 (*n* = 22), KLHL1–KO: V_50_ = –44.3 ± 0.9 mV* and *k* = 5.1 ± 0.3 (*n* = 27). *P < 0.05, *t*-test, *t* = 2.090 (df = 47). SSI values for WT neurons: V_50_ = –65.4 ± 1.0 mV, *k* = 7.1 ± 0.5 (WT, *n* = 21); KLHL1 KO: V_50_ = –67.5 ± 0.7 mV* and *k* = 6.1 ± 0.2* (*n* = 27). *P < 0.05, *t*-test, *t* = 1.736 for V_50_ and *t* = 2.251 for *k*, df = 46.

Biophysical analysis of T-type calcium currents (Figure 3) depicts changes consistent with an overexpression of the α_1__G_ component (Ca_V_3.1) in KLHL1-KO neurons. As seen in Figure 3A, the rise time of activation from –40 to –20 mV was significantly smaller (faster); for instance, the rise time at −40 mV was 10.9 ± 0.9 ms in WT compared to 7.9 ± 0.6 ms in KLHL1-KO neurons (*P* < 0.05, *t*-test). The time constant of inactivation was different at two voltages only (−30 and −40 mV (WT, 33.9 ± 2.7 ms *vs.* KO, 25.5 ± 2.2 ms at −40 mV), Figure 3B; *P* < 0.05, *t*-test). Recovery from inactivation (RFI) was faster in KLHL1-KO neurons (τ_W, KLHL__1__–KO_ = 204 ± 15 ms *vs.* τ_W, WT_ = 305 ± 33 ms, *P* < 0.05, *t*-test, Figure 3C). The current deactivation time constant was slower in KLHL1-KO neurons (Figure 3D) at all ranges tested (e.g., τ_deactivation__, –__60__ mV_ was 4.8 ± 0.4 ms for WT and 6.4 ± 0.5 ms for KLHL1-KO neurons). Lastly, steady-state analysis shows that V_50_ values for activation (SSA) and inactivation (SSI) were both discretely but significantly shifted ∼−2 mV in KLHL1-KO compared to WT neurons (Figure 3E; *P* < 0.05, *t*-test). SSA V_50_ was −41.8 ± 0.7 mV in WT (Perissinotti et al., 2021) compared to −44.3 ± 0.9 mV in KLHL1-KO (*P* < 0.05, *t*-test); no change in the slope factor *k* was observed (5.2 ± 0.3 for WT and 5.1 ± 0.3 for KO). Comparably, SSI V_50_ changed from −65.4 ± 1.0 mV in WT (Perissinotti et al., 2021) to −67.5 ± 0.7 mV in KO (*P* < 0.05, *t*-test), and the slope factor *k* changed from 7.1 ± 0.5 to 6.1 ± 0.2 (*P* < 0.05, *t*-test). Accordingly, the changes in the steady-state activation and inactivation curves in KLHL1-KO neurons resulted in a ∼−4 mV shift in the cross point of the window currents (shaded area under the steady-state curves, Figure 3D).

### Intrinsic Properties of KLHL1-KO Hypothalamic Neurons

**FIGURE 4:**
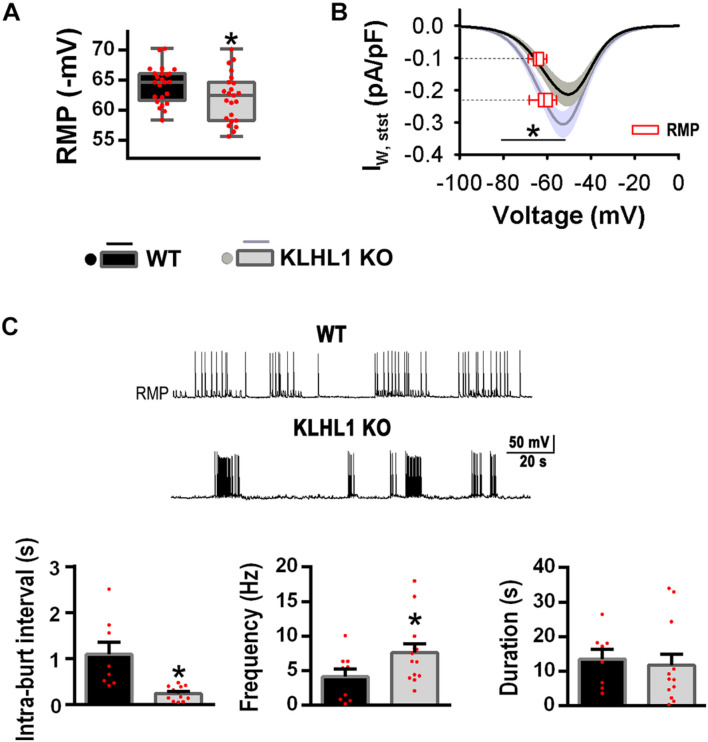
Increased T-type window currents and burst-firing patterns in KLHL1-KO neurons. **(A)** Resting membrane potential (RMP) in WT (*n* = 24) and KLHL1-KO (*n* = 23) neurons. *P < 0.05, *t*-test, *t* = 2.120 (df = 45). **(B)** T-type steady-state currents in WT (*n* = 18) and KLHL1-KO (*n* = 23) neurons. Window currents were calculated for each recording neuron from the SSA and SSI curves fitted to a Boltzmann function; SEM is indicated in gray. The steady-state current at the RMP (empty red box plot) is indicated for both genotypes. *P < 0.05, Two Way ANOVA, *F*_1_,_139_ = 13.468 (from –50 to –80 mV). There is a statistically significant interaction between the genotype and the voltage (*F*_11_,_420_ = 2.300) (from –20 to –80 mV). **(C) Top:** Example of firing patterns in WT and KLHL1-KO neurons. **Botton:** Mean-intraburst interval, frecuency and duration of the burst activity. *P < 0.05, *t*-test, *t* = 3.770 for mean-intraburst interval and *t* = 1.900 for frequency (df = 18, *n* = 8 for WT, *n* = 12 for KLHL1 KO).

As shown in Figure 4A, the RMP of KLHL1-KO neurons was depolarized by ∼2 mV compared to WT (*P* < 0.05, *t*-test). We calculated the T-type window current using the steady-state activation and inactivation curves fitting parameters (Bijlenga et al., 2000; Lambert et al., 2014; Perissinotti et al., 2021; Rivero-Echeto et al., 2021). Figure 4B depicts T-type window currents as function of voltage. The superimposed dashed lines show interpolated values for RMP (Y-axis), pinpointing the T-type steady-state current at the respective RMP. Steady-state window currents displayed a leftward shift in KLHL1-KO neurons (*P* < 0.05, Two-way ANOVA). Noticeably, the steady-state calcium current at the RMP was ∼2.3 times larger for KLHL1-KO than WT neurons.

The presence of window currents is related to spontaneous firing pattern and neuronal excitability; indeed, the percentage of POMC neurons displaying spontaneous firing of action potentials was 31% (10 of 35) in WT compared to 54% (21 of 39) in KLHL1-KO (*P* < 0.05, Z-test). The firing pattern was different in KO neurons, displaying lower mean-intraburst interval (1.1 ± 0.3 s for WT *vs*. 0.5 ± 0.1 s for KO) and higher intra-burst frequency compared to WT (*P* < 0.05, *t*-test). No significant difference in the burst duration was observed between genotypes (*P* < 0.05, *t*-test) (Figure 4C).

**FIGURE 5:**
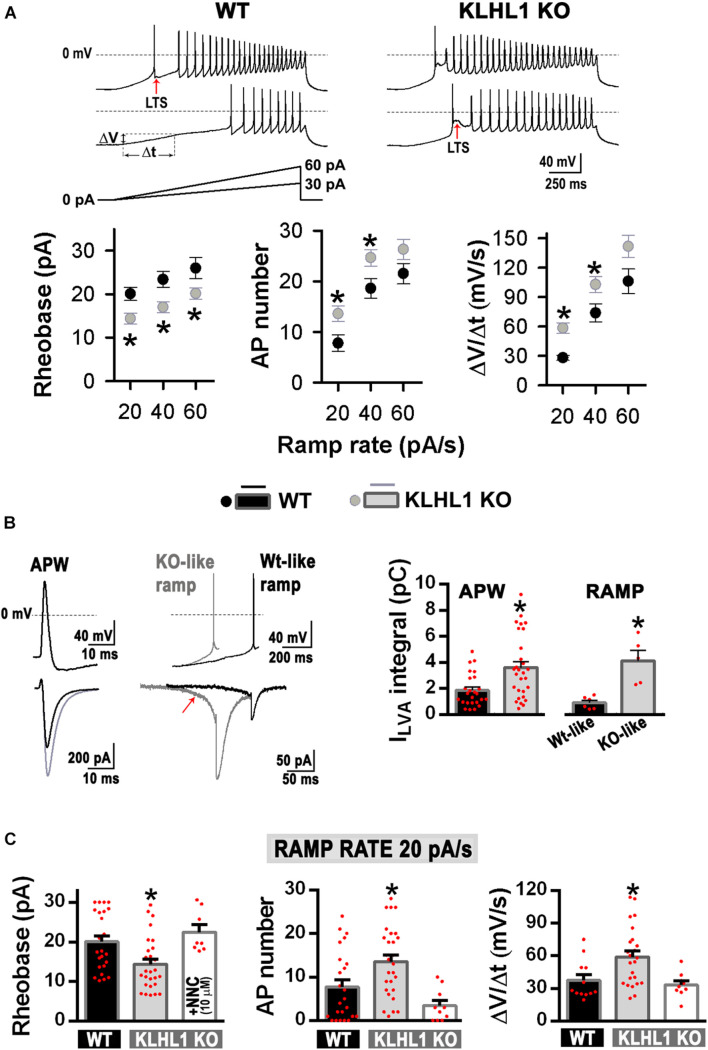
Increased membrane excitability in KLHL1-KO neurons. **(A) Top:** Examples of neuronal firing elicited from V_H_ = –75 mV by a 20 and 40 pA/s ramps in WT and KLHL1-KO neurons. Red arrows indicate low-threshold spike (LTS) responses. **Bottom:** Rheobase (pA), number of action potentials and rate of membrane potential depolarization (Slope = ΔV/Δ*t*, mV/s, see panel A in response to 20, 40 and 60 pA/s ramps) in WT (*n* = 24, 24, 12) and KLHL1-KO (*n* = 28, 28, 24) neurons. *P < 0.05, *t*-test. Rheobase: *t* = 2.924 for 20 pA/s, *t* = 3.107 for 40 pA/s, *t* = 2.155 for 60 pA/s, df = 50. AP number: *t* = 2.607 for 20 pA/s, *t* = 2.428 for 40 pA/s, df = 50. Slope: *t* = 2.481 for 20 pA/s, *t* = 2.108 for 40 pA/s, df = 34. **(B) Left:** Average traces of T-type LVA currents elicited by an action potential waveform (ΔV/Δ*t* = 0 mV/s, APW protocol). **Center:** Isolated LVA component traces elicited by action potentials preceded by a slope and a rheobase representative of either WT (WT-like ramp, ΔV/Δ*t* = 30 mV/s, rheobase = 20 pA) or KLHL1-KO neurons (KO-like ramp, ΔV/Δ*t* = 60 mV/s, rheobase = 14 pA). The KO-like ramp protocol favors T-type channel recruitment (red arrow). **Right:** Average LVA current integral in WT and KLHL1-KO neurons. *P < 0.05, *t*-test. APW protocol: *t* = 3.241 (df = 52, *n* = 25 for WT, *n* = 29 for KLHL1 KO). WT- or KO-like ramp protocols: *t* = 4.372 (df = 9, *n* = 6 for WT, *n* = 5 for KLHL1 KO). **(C)** Quantification of rheobase (pA), number of action potentials and rate of membrane potential depolarization (Slope = ΔV/Δ*t*, mV/s) in WT (*n* = 24, 24, 12), KLHL1-KO (*n* = 28, 28, 24) and NNC 55-0396 (10 μM)-treated KLHL1-KO (*n* = 8, 10, 9) neurons in response to 20 pA/s ramps. *Significantly different from WT and NNC-treated KLHL1-KO neurons. *P* < 0.05, ANOVA. Rheobase: F_2_,_57_ = 6.743, AP number: F_2_,_59_ = 8.004, Slope: F_2_,_42_ = 6.029.

Membrane excitability was assesed using depolarizing current ramps (20, 40, and 60 pA/s) from a preset membrane potential of ∼–75 mV to circumvent spontaneous firing. Of note, cell capacitance, input resistance and the shape of single APs were indistinguisable between WT and KLHL1-KO neurons (Table 1). Figure 5A shows examples of the AP firing evoked at 20 and 40 pA/s ramp rates in WT and KLHL1-KO neurons. Note that a large low-threshold spike (LTS) response was elicited by the 20 pA/s ramp rate in KLHL1-KO neurons. In contrast, a faster ramp rate (40 pA/s) was necessary to evoke such response in WT neurons. A LTS response refers to a large membrane depolarization mediated by an increase of T-type calcium conductance. These results are in line with the high T-type current density observed in KLHL1-KO cultures (Figures 2–4). To quantitatively analyze the voltage responses to the current ramps, three different measurements were calculated: the rheobase, the number of APs triggered, and the rate of membrane potential depolarization preceding the first AP (i.e., the slope of depolarization, ΔV/Δ*t*) (Figure 5A) (Perissinotti et al., 2021). All the data indicates increase excitability in KLHL1 KO neurons. The rheobase was ∼25% lower in KLHL1-KO neurons than WT across all ramp rates tested (*P* < 0.05, *t*-test). Similarly, the number of action potentials was increased by ∼50% at ramp rates of 20 and 40 pA/s in KLHL1-KO neurons (*P* < 0.05, *t*-test). In addition, the rate of membrane depolarization was ∼2 times faster in KLHL1-KO than WT mice (58.3 ± 5.3 *vs*. 28.0 ± 2.6 mV/s for a ramp rate of 20 pA/s; *P* < 0.05, *t*-test).

**TABLE 1:** Passive and active properties of WT and KLHL-1 KO hypothalamic neurons.

	WT	KLHL1 KO
**Passive properties**		
*R*_in_ (GΩ)	1.52 ± 0.10(47)	1.40 ± 0.09(45)
*C*_*m*_ (pF)	24.5 ± 2.2(26)	26.8 ± 2.0(28)
**Action potential**		
Peak (mV)	42.1 ± 3.9(12)	49.5 ± 2.7(12)
Duration at base (ms)	8.3 ± 0.7(12)	10.2 ± 1.4(12)
Rise time (ms)	5.2 ± 0.5(12)	4.9 ± 0.2(12)
Fall time (ms)	3.1 ± 0.3(12)	5.3 ± 1.5(12)
AHP depth below V_m_ (mV)	5.9 ± 0.8(12)	6.1 ± 1.3(12)
AHP duration to 50% (ms)	72.2 ± 19.0(12)	54.9 ± 15.8(12)

*AHP, after-hyperpolarization. The input resistance (R_in_), the capacitance (C_m_) and the shape of single action potential in hypothalamic neurons were not significantly different between genotypes. P > 0.05, t-test.*

Due to the unique biophysical properties of T-type currents, their contribution is noticeable during an action potential, as seen in Figure 5B (left trace), where the LVA calcium influx in response to an action potential waveform (APW) was 72% larger in KLHL1-KO neurons (*P* < 0.05, *t*-test). Faster rates of membrane depolarization favor T-type channel activity because they induce less current inactivation prior to activation of an action potential (Gutierrez et al., 2001). To determine whether differences in the T-type channel recruitment yield the observed alterations in membrane excitability between genotypes, we determined LVA calcium influx in: (1) WT neurons during an AP preceded by a slow rate of membrane depolarization with a rheobase similar to that observed in WT neurons in response to a ramp rate of 20 pA/s (“WT-like ramp,” ΔV/Δ*t* ∼30 mV/s, rheobase ∼20 pA), and (2) KLHL1-KO neurons during an AP preceded by a fast rate of membrane depolarization with a rheobase similar to that observed in KLHL1-KO neurons in response to a ramp rate of 20 pA/s (“KO-like ramp,” ΔV/Δ*t* ∼60 mV/s, rheobase ∼14 pA) (Figure 5B). The KO-like ramp protocol favored T-type channel recruitment (red arrow) and produced greater LVA calcium influx during an action potential in KLHL1-KO neurons. In contrast, a WT-like protocol recruited less T-type channels, producing a small LVA-mediated calcium influx during an action potential in WT neurons. In average, KLHL1-KO neurons displayed ∼4.5 times more LVA calcium influx than WT neurons (*P* < 0.05, *t*-test, Figure 5B, right panel). Furthermore, application of micromolar concentrations of T-type blocker NNC 55-0396 (10 μM) to KLHL1-KO neurons increased the rheobase level, slowed down the rate of membrane depolarization and reduced the triggered number of APs in response to a ramp rate of 20 pA/s, comparable to WT neurons (*P* < 0.05, *t*-test, Figure 5C).

### Leptin Resistance in KLHL1-KO Neurons

So far, we found T-type channel overexpression in KLHL1-KO hypothalamus leads to increases currents and membrane excitability in POMC neurons. It is well-established that POMC neurons increase their excitability in response to administration of nanomolar levels of leptin through the activation of TRPC channels (Qiu et al., 2010, 2011, 2014). We recently reported that TRPC1/5-Ca_V_3.1/2 complexes mediates leptin-induced excitability in WT POMC neurons (Perissinotti et al., 2021). Therefore, we next analyzed the leptin-mediated effects on membrane excitability on KLHL1-KO hypothalamic neurons.

**FIGURE 6:**
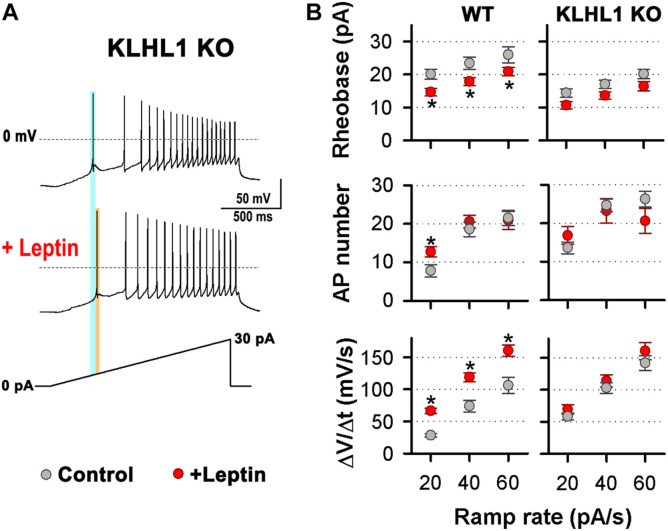
Electrical Resistance to leptin in KLHL1 KO neurons. **(A)** Examples of neuronal firing elicited from V_H_ = –75 mV by a 20 pA/s ramp in control and leptin-treated (100 nM) KLHL1-KO neurons. Light blue and orange lines indicate the rheobase before and after the addition of leptin, respectively. **(B)** Left: Rheobase (pA), number of action potentials and rate of membrane potential depolarization (Slope = ΔV/Δ*t*, mV/s) in response to 20, 40 and 60 pA/s ramps in KLHL1 KO control (*n* = 28, 28, 25) and leptin-treated (*n* = 14, 14, 13) neurons. WT neuron behavior is shown for comparison (from Perissinotti et al., 2021). *Significantly different from control, *P* < 0.05, *t*-test.

**FIGURE 7:**
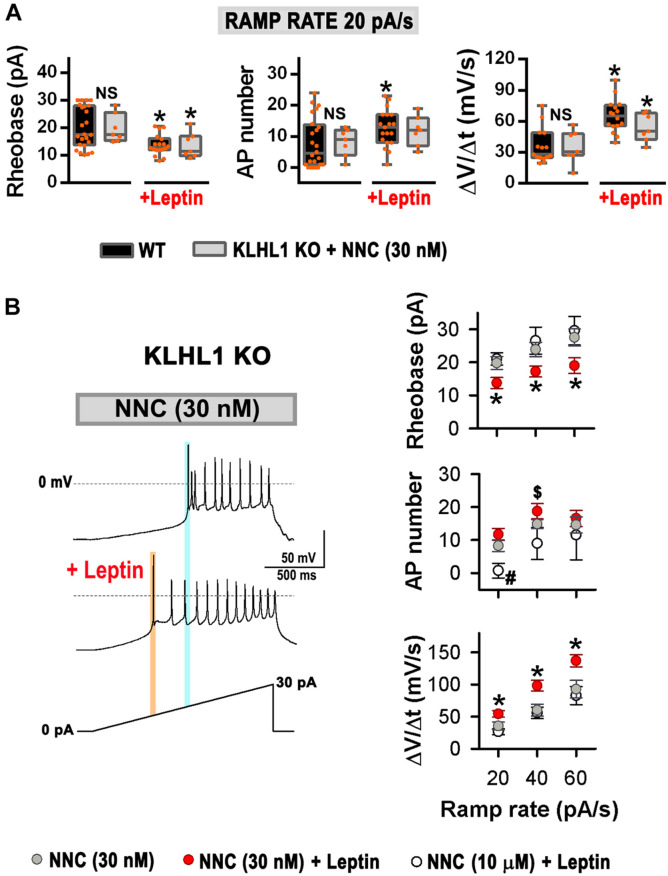
Resistance to leptin in KLHL1 KO neurons is restored by partial blockade of T-type currents. **(A)** Leptin effect on rheobase (pA), number of action potentials and rate of membrane potential depolarization (Slope = ΔV/Δ*t*, mV/s) in WT (control, *n* = 24, 24, 15; + leptin, *n* = 18, 19, 16) and [NNC (30 nM)]-treated KLHL1-KO (control, *n* = 7, 7, 7; + leptin, *n* = 7, 7, 7) neurons in response to a ramp rate of 20 pA/s. NS, no significant difference between WT and [NNC (30 nM)]-treated KLHL1-KO, *P* > 0.05, *t*-test. *Significantly different from its respective control), *P* < 0.05, *t*-test. WT: Rheobase: *t* = 3.285 (df = 40), AP number: *t* = 2.257 (df = 41), Slope: *t* = 5.059 (df = 29). [NNC (30 nM)]-treated KLHL1-KO: Rheobase: *t* = 2.368 (df = 12), Slope: *t* = 2.362 (df = 12). **(B) Left:** Examples of neuronal firing in KLHL1-KO neurons preincubated with NNC (30 nM) before and after the addition of leptin [NNC (30 nM) + leptin (100 nM)]. V_H_ = 75 mV, AP triggering was elicited by a 20 pA/s ramp. Light blue and orange lines indicate the rheobase in the NNC treatment and after the addition of leptin ([NNC + leptin] treatment). **Right:** Rheobase (pA), number of action potentials and rate of membrane potential depolarization (Slope = ΔV/Δ*t*, mV/s) in response to 20, 40 and 60 pA/s ramps in [NNC (30 nM)]-treated (*n* = 7), [NNC (30 nM) + leptin]-treated (*n* = 7) and [NNC (10 μM) + leptin]-treated (*n* = 5) KLHL1-KO neurons. *Significantly different from NNC (30 nM) and [NNC (10 μM) + leptin]. ^#^Significantly different from NNC (30 nM) and [NNC (30 nM) + leptin]. ^$^Significantly different from [NNC (10 μM) + leptin]. *P* < 0.05, ANOVA. Rheobase: *F*_2_,_16_ = 3.867 for 20 pA/s, *F*_2_,_16_ = 4.188 for 40 pA/s and *F*_2_,_16_ = 3.648 for 60 pA/5. AP number: *F*_2_,_16_ = 8.550 for 20 pA/s, *F*_2_,_16_ = 4.081 for 40 pA/s. Slope: *F*_2_,_16_ = 5.908 for 20 pA/s, *F*_2_,_16_ = 6.832 for 40 pA/s, *F*_2_,_14_ = 4.770 for 60 pA/s.

Leptin induces increased membrane excitability in response to ramp rates of 20, 40 and 60 pA/s in WT neurons (Perissinotti et al., 2021). Remarkably, using the same ramp protocol, no differences in the rheobase, the number of APs and the rate of membrane depolarization were observed between untreated neurons (control) and leptin-treated KLHL1-KO neurons (Figures 6A,B); suggesting that the excitability in KLHL1-KO neurons may have reached a plateau under basal conditions. To test this assumption, we studied whether decreasing excitability of KLHL-KO neurons could restore leptin sensitivity. T-type channels were partially inhibited with low concentration of NNC 55-0396 (30 nM) to decrease their membrane excitability. The resulting rheobase, AP number and membrane depolarization rate of KLHL1-KO neurons were indistinguishable from WT neurons (*P* > 0.05, *t*-test, Figure 7A). Moreover, this partial blockade of T-type currents restored leptin sensitivity to similar levels to those observed in WT neurons (*P* < 0.05, *t*-test, Figure 7A). Figure 7B shows leptin effects on membrane excitability in response to ramp rates of 20, 40 and 60 pA/s in [NNC (30 nM)]-treated KLHL1-KO neurons. Leptin increased membrane excitability across all ramps tested. As expected, further application of micromolar concentrations of NNC 55-0396 (10 μM) prevented leptin-mediated effects on membrane excitability, confirming the role of LVA channels in this process. Overall, the data show the electrical efficacy of leptin depends on the neuron’s basal excitability level; the larger T-type LVA current activity seen in KLHL1-KO POMC neurons increases their excitability to a plateau level which cannot be further enhanced by the presence of leptin; causing electrical resistance to leptin (i.e., loss of electrical sensitivity lo leptin) that is completely reversible upon partial blockade of T-type channels.

### Systemic Deletion of KLHL1 Results in Increased Body Weight and Altered Food Ingestion

**FIGURE 8:**
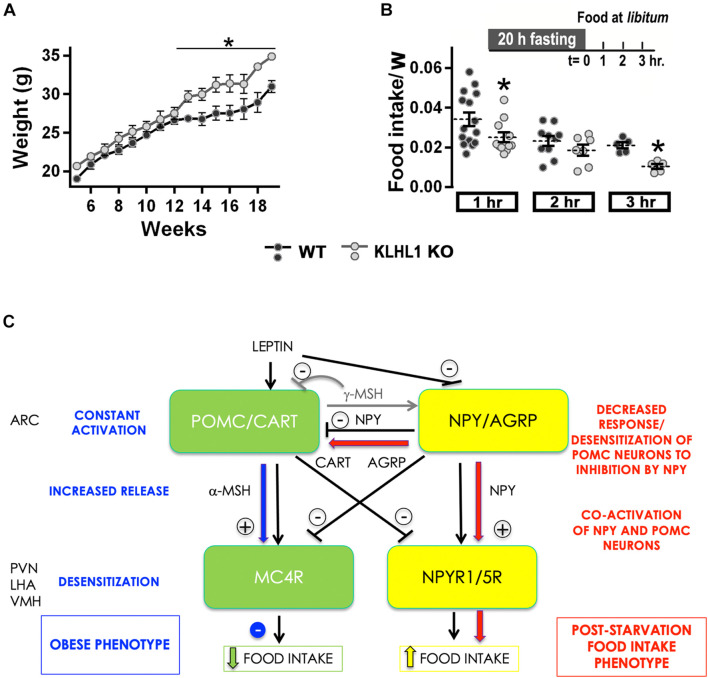
Increased body mass and abnormal fasting response in KLHL1 KO mice. **(A)** Body mass plotted against mice age for WT (black circles, *n* = 4–10) and KLHL1 KO (gray circles, *n* = 4–9). *Significantly different after the 12th week, *P* < 0.05, Two-Way ANOVA, F_1_,_183_ = 58.84. **(B)** Fasting experiment protocol: adult male WT and KLHL1-KO mice were fasted for 20 h. Weight gain and food consumption were examined hourly after food reintroduction (*t* = 0) for three hours. Food intake normalized by weight ratio was calculated after food reintroduction: 1h (WT, KO; *n* = 15, 11), 2h (WT, KO; *n* = 10, 7) and 3h (WT, KO; *n* = 5, 5). *P < 0.05, *t*-test; 1h: *t* = 2.004, df = 24; 3h: *t* = 5.451, df = 8. **(C)** *Reciprocal control of satiety and feeding centers:* The black arrows depict a simplified version of the reciprocal control occurring between the feeding (yellow) and the satiety (green) circuits in hypothalamic nuclei involved in the regulation of energy homeostasis: Arcuate (ARC), paraventricular (PVN) and ventromedial (VMH) and lateral hypothalamic area (LHA). The hyper-excitable and increased depolarized state of POMC neurons could counteract the inhibitory action of NPY, permitting the activation of both satiety and feeding centers, dampening the food intake post-starvation (red). On the other hand, continued hyper-excitability in KLHL1 KO POMC neurons would lead to increased α-MESH release onto MC4R neurons, likely causing long-term desensitization and impairing the satiety response, which would contribute to the obesity phenotype (blue).

Leptin is a key hormone in the regulation of energy balance and plays an essential role in energy homeostasis (Ahima et al., 1996). We assessed the body mass changes of KLHL1-KO mice with age, they displayed significant increases compared to WT after week 12 (Figure 8A, *P* < *0.05*, Two-way ANOVA) suggesting abnormal energy intake/expenditure balance. We used a starvation-refeeding paradigm as a quick appraisal of whether the mice would respond normally to increased levels of leptin after refeeding. We performed a 20-hr fasting experiment followed by exposure to normal chow at *libitum* (Figure 8B; Scott et al., 2009; Liu et al., 2012). Pre-, and post-fasting weight after food reintroduction was monitored for three hours. Both mice cohorts lost similar amounts of weight after fasting (∼10%, not shown). As expected, WT mice displayed sizable food ingestion during the first hour exposed to nourishment, which tapered by the second and third hours exposed to food *ad libitum*, indicating a normal anorexigenic response to leptin. However, when presented with food at *libitum*, food consumption in the KLHL1-KO cohort was slower than in WT mice (Figure 8B, *P* < *0.05*, *t*-test), suggesting that KLHL1-KO mice were less sensitive to starvation, displaying abnormal hunger responses.

## Discussion

This study provides new insights into the importance of T-type channels in the leptin signaling cascade leading to membrane excitability in POMC hypothalamic neurons. We previously reported that TRPC1/5-Ca_V_3.1/2 complexes mediate leptin-induced excitability in POMC neurons (Perissinotti et al., 2021), here we show that over-expression of the pore-forming α_1__G_ subunit of the T-type Ca_V_3.1 channel in the KLHL1-KO mice altered their basal excitability, rendering them electrically unresponsive to leptin. Leptin responses were fully restored by partial blockade of T-type channels. Neither leptin receptor (LRb) nor downstream effectors (STAT3, PI3K phosphorylation, TRPC1/5 channels) were altered in the KLHL1-KO hypothalamus.

T-type calcium channels are widely distributed in the hypothalamus (Qiu et al., 2006). mRNA and protein levels of the α_1__G_ subunit of the Cav 3.1 channel are highly expressed; whereas α_1__H_ and α_1__I_ subunits (of Cav 3.2 and Cav 3.3 channels, respectively) are also present but at much lower levels (Craig et al., 1999; Talley et al., 1999). The role of T-type channels is well established, they are expressed in several nuclei and help control neuronal excitability. ARC neurons containing neuropeptide Y (NPY) and agouti-related peptide (AgRP) are conditional pacemakers. T-type currents drive oscillatory activity of NPY/AgRP neurons, in which burst firing frequency is modulated by a transient outwardly rectifying potassium conductance (van den Top et al., 2004). The leptin-dependent inhibition of AgRP/NPY neurons is attributed in part to modulation of K^+^ channels such as K_*ATP*_, BK and K_V_2.1 channels (Spanswick et al., 1997; Mirshamsi et al., 2004; Yang et al., 2010; Baver et al., 2014). The orexigenic peptide Ghrelin is reported to directly activate a sustained depolarizing conductance with the pharmacologic properties of SUR1/Trpm4 non-selective cation channels (sulfonylurea receptor 1/transient receptor potential melastatin 4), which activates T- and R-type voltage dependent Ca^2+^ channels in NPY/AgRP neurons (Hashiguchi et al., 2017). Notably, both leptin-dependent activation of POMC neurons and ghrelin-dependent activation of AgRP/NPY neurons involves the subsequent activation of T-type channels to increase neuronal activity (Qiu et al., 2006, 2018; Bosch et al., 2009; Hashiguchi et al., 2017; Perissinotti et al., 2021).

In line with the large hypothalamic Ca_V_3.1 channel expression, T-type current density was also increased in POMC neurons in KLHL1-KO mice. Biophysical analysis of T-type currents is consistent with overexpression of the α_1__G_ component (Perez-Reyes, 2003). Our lab demonstrated that KLHL1 protein positively modulates α_1__H_ subunit of the Ca_V_3.2 channel but it does not affect Ca_V_3.1 nor Ca_V_3.3 in the hippocampus (Perissinotti et al., 2015) or in HEK cells that constitutively overexpress these isoforms (Aromolaran et al., 2010). The increased Ca_V_3.1 expression seen in the hypothalamus is likely the result of homeostatic compensation when KLHL1 is absent, although the molecular mechanisms responsible are currently being investigated.

The biophysical properties of T-type channels permit their stimulation by small depolarizations near the resting potential, rendering them optimal for regulating membrane excitability and burst-firing pattern under physiological conditions near resting state. Accordingly, changes in the steady-state activation and inactivation curves in KLHL1-KO neurons resulted in a ∼-4 mV shift in the window current cross point. This leftwards shift led to a 90% increase of the steady-state calcium current in KLHL1-KO neurons at the resting membrane potential, which was ∼2 mV more depolarized than WT neurons. Intrinsic neuron properties such as the spontaneous firing and the basal excitability are tightly related to T-type “window currents” (Huguenard, 1996; Perez-Reyes, 2003; Crunelli et al., 2005; Molineux et al., 2006; Bayazitov et al., 2013); fittingly, we found increased burst-firing pattern and excitability in POMC neurons from the KLHL1-KO mice. In line with our results, it has been reported that the knockdown of Ca_V_3.1 calcium channel switch from bursting to tonic firing at thalamocortical projections (Bayazitov et al., 2013). Interestingly, our results demonstrate that changes that alter the basal excitability of POMC neurons, such as over-expression of Ca_V_3.1 T-type channels, rendered them unresponsive to leptin. Partial blockade of T-type channels brought KLHL1-KO neurons to normal excitability levels, which was sufficient to restore leptin sensitivity in these neurons.

Energy imbalance alters Ca^2+^ handling and excitability of POMC neurons (Paeger et al., 2017). Intracellular Ca^2+^ levels are regulated by several mechanisms including plasma membrane ion channels (e.g., voltage-gated and ligand-gated Ca^2+^ channels), ion exchangers and “pumps,” as well as the release from the intracellular Ca^2+^ stores (Verkhratsky and Toescu, 2003). Correspondingly, endoplasmic reticulum (ER) stress was recognized as an important causal factor for the development of leptin resistance induced by diet (Hosoi et al., 2008; Ramírez and Claret, 2015). Diet-induced obesity (DIO) causes increased resting cytosolic Ca^2+^ levels and markedly decreased excitability of anorexigenic POMC neurons, resulting from increased calcium-activated K^+^ channel activity (SK channels) (Paeger et al., 2017). The overexpression of Ca_V_3.1 reported here could lead to increased membrane permeability to calcium at rest due to T-type channel’s window current properties (Chemin et al., 2000). However, this is an unlikely scenario (or it is not physiologically relevant in our system), as we have shown that Ca^2+^ influx through T-type channels contributes locally at the submembrane level and not globally to support increased excitability in POMC neurons (Perissinotti et al., 2021).

Although our systemic KLHL1-KO model is not a good model to explore questions regarding obesity and altered starvation responses changes observed in these mice, we examined the possible contribution of the altered molecular mechanisms observed in the KLHL1 KO to these phenotypes. Upregulation of Ca_V_3.1 channels could contribute to obesity (Kortekaas and Wadman, 1997; Yunker et al., 2003; Aguado et al., 2016). Deletion of Ca_V_3.1 channels (Cacna1g KO mice) or treated with TTA-A2 (selective blocker of T-type Ca_V_3) results in reduced food intake, attenuated weight gain and decreased body fat gain in response to high-fat diet; suggesting a role for Ca_V_3.1 channels in weight maintenance (Uebele et al., 2009; Rosenstand et al., 2020). Likewise, ethosuximide, another T -type calcium channel blocker, partially protects against neurosteroid-induced obesity in mice by reducing food intake and body weight (Chidrawar and Ali, 2020); similarly, T-type antagonists prescribed for epilepsy, depression, obsessive-compulsive disorder and bulimia nervosa can cause loss of appetite as a side effect (Cookson and Duffett, 1998; Traboulsie et al., 2006; Willfong and Willmore, 2006). Ca_V_3.1 channels have been proposed as potential therapeutic targets for the prevention and treatment of obesity (Uebele et al., 2009; Vasconcelos et al., 2016; Chidrawar and Ali, 2020; Zhai et al., 2020).

Furthermore, we found that the fasting-induced response to hunger was abnormal in KLHL1-KO mice, they consumed food at a slower rate than WT mice after twenty hours of food deprivation (short-term). Similarly, mice that exhibit obese phenotypes [ob/ob and diet-induced obesity (DIO)] consumed less food compared to WT mice in response to twenty-four hours food deprivation (Ueno et al., 2007). Factors contributing to the dampened response to hunger in obese mice could be explained by the presence of large readily available fat stores that can be mobilized when food is deprived (Ramirez and Sprott, 1978). Alternatively, it can also be explained by a decreased sensitivity to fasting-induced orexigenic signals or by an increased sensitivity to satiety-induced anorexigenic signals (Ueno et al., 2007). Satiety and anorexigenic signals activate POMC neurons while they inhibit NPY/AgRP neurons; in contrast, starvation and orexigenic signals activate NPY/AgRP neurons and inhibit POMC neurons (Figure 8C; Schwartz et al., 1996; Elias et al., 1999; Cowley et al., 2001). The cross-regulation between POMC and AgRP neurons is complex (Figure 8C), for instance, the release of alpha-melanocyte stimulating hormone (α-MSH) -a potent anorexigenic in central melanocortin receptor 4 (MC4R) neurons- is promoted by satiety signals, while it is inhibited by starvation signals (reviewed by Minokoshi, 2020). Leptin causes satiety by triggering POMC neuron depolarization and production and release of α-MSH from axon terminals, which activates MC4R neurons and result in suppressed food intake and increased energy expenditure (Cowley et al., 2001; Guo et al., 2004; O’Rahilly et al., 2004; D’Agostino and Diano, 2010; Qiu et al., 2010; Anderson et al., 2016; Baldini and Phelan, 2019). Our data shows this pathway is constitutively activated in cultured KLHL1-KO POMC neurons due to their hyper-excitable basal status and increased burst-firing pattern. Activity patterning is very important in hypothalamic neuron neuropeptide release, and burst firing is a more effective stimulus for peptide release than fast, repetitive firing patterns of activity (Poulain and Wakerley, 1982). Thus, increased spontaneous burst-firing pattern (intra-burst frequency) in KLHL1-KO POMC neurons could enhance basal release of α-MSH from axon terminals, eventually leading to MC4R desensitization and constitutive, partially active satiety responses that would contribute to the obesity phenotype (Figure 8C, blue arrows; Gao et al., 2003; Shinyama et al., 2003; Mohammad et al., 2007; McDaniel et al., 2012; Granell et al., 2013).

On the other side, putative AgRP^+^/NPY^+^ neurons from KLHL1 KO mice elicit normal responses to leptin (decreased excitability), thus we hypothesize that activation of the feeding center and NPY release would result in normal food intake post-starvation if successful feedback inhibition of POMC neurons occurred. Application of 100 nM NPY leads to a ∼17 mV average hyperpolarization of POMC neurons, which efficiently prevents triggering of APs (Roseberry et al., 2004). However, the slightly depolarized state of KLHL1 KO POMC neurons and increased T-type channel window current activity could partially counteract the inhibitory effect of NPY, resulting in incomplete activity of the satiety circuit even after food deprivation. Moreover, increased inhibitory stimulus onto POMC neurons by NPY could also lead to long-term desensitization of this inhibitory feedback loop. Here we assessed the acute response of KLHL1 KO mice to starvation; thus, it is possible that activation of the feeding center in parallel to partial activation of the satiety center contributes to the dampened feeding response seen under our experimental conditions (Figure 8C, red arrows). Interestingly, Hashiguchi et al. (2017) reported T-type channels as targets in the activation pathway of NPY/AgRP in response to orexigenic signals. Further studies are necessary to determine whether Ca_V_3.1 channels are upregulated in NPY/AgRP neurons and possible implications on their excitability.

In summary, increased excitability in KLHL1-KO POMC neurons induced the loss of electrical sensitivity lo leptin, contributing to alteration of the hypothalamic feeding and satiety neuron network dynamics that caused dysregulating the energy balance and the body weight. This work corroborates that T-type currents are indispensable in POMC neuron excitability, which is essential for transduction of the leptin cascade. Increased POMC neuron excitability due to increased Ca_V_3.1 T-type channel activity in the KLHL1-KO mice resulted in electrical resistance to leptin, which could be rescued by simply reducing T-type channel activity.

## Data Availability Statement

The raw data supporting the conclusions of this article will be made available by the authors, without undue reservation.

## Ethics Statement

The animal study was reviewed and approved by Institutional Animal Care and Use Committee at Loyola University Chicago (IACUC 2016032).

## Author Contributions

EP-R conceived of the study, supervised the experiments, and approved the final version of the manuscript. EP-R, EM-H, and PP designed the experiments, performed the experiments, analyzed the data, interpreted the results of experiments, and prepared the figures. YH and MK generated the KLHL1-knockout mice. EP-R and PP wrote the manuscript, edited and revised the manuscript. All authors contributed to the article and approved the submitted version.

## Conflict of Interest

The authors declare that the research was conducted in the absence of any commercial or financial relationships that could be construed as a potential conflict of interest.

## Publisher’s Note

All claims expressed in this article are solely those of the authors and do not necessarily represent those of their affiliated organizations, or those of the publisher, the editors and the reviewers. Any product that may be evaluated in this article, or claim that may be made by its manufacturer, is not guaranteed or endorsed by the publisher.

## Abbreviations

AgRP: agouti-related peptide
DIV: days *in vitro*
KLHL1: Kelch-like 1
LRb: leptin receptor
LVA: low-voltage activated
HVA: high-voltage activated
LTS: low-threshold spike
NPY: neuropeptide Y
POMC: hypothalamic proopiomelanocortin positive neurons
TRPC: transient receptor potential cation.
